# Predictors of Postpartum Post-Traumatic Stress Disorder Following Traumatic Birth: The Influence of Lifetime Trauma, Violence, and Coping Strategies—A Prospective Study

**DOI:** 10.3390/nursrep15120420

**Published:** 2025-11-28

**Authors:** Eirini Orovou, Antigoni Sarantaki, Vaidas Jotautis, Zacharias Kyritsis, Maria Tzitiridou Chatzopoulou

**Affiliations:** 1 Department of Midwifery, University of Western Macedonia, 50200 Ptolemaida, Greece; 2 Department of Midwifery, University of West Attica, Agioy Spyridonos 28, 12243 Athens, Greece; esarantaki@uniwa.gr; 3 Faculty of Medicine, Kauno Kolegija Higher Education Institution, Pramonės pr. 20, 50468 Kaunas, Lithuania; vaidas.jotautis@go.kauko.lt; 4 School of Mathematics, Aristotle University of Thessaloniki, 54124 Thessaloniki, Greece; zkyrit@math.auth.gr

**Keywords:** postpartum PTSD, traumatic birth, emergency caesarean section, operative vaginal delivery, domestic violence, trauma, coping strategies, anxiety

## Abstract

**Background/Objectives**: Childbirth, although generally a positive life event, can sometimes be experienced as traumatic, leading to postpartum post-traumatic stress disorder. Emergency caesarean section and operative vaginal delivery are associated with elevated psychological distress, while factors such as lifetime trauma, domestic violence, anxiety, and coping strategies may further increase vulnerability. **Methods**: This prospective cohort study included 113 postpartum women who delivered via emergency caesarean section (73.5%) or operative vaginal delivery (26.5%) in two tertiary hospitals in Athens, Greece (March–July 2023). Data were collected at three time points: the second postpartum day, six weeks postpartum, and three months postpartum. Descriptive statistics were used to summarize sample characteristics. Chi-square tests were performed for categorical variables and independent sample t-tests for continuous variables. Multivariate logistic regression analyses were conducted to identify predictors of postpartum post-traumatic stress disorder, with results expressed as odds ratios (OR) and 95% confidence intervals (CI). **Results**: At six weeks postpartum, 14.2% of participants met full diagnostic criteria for P-PTSD. Postpartum post-traumatic stress was strongly associated with higher state and trait anxiety, fewer positive coping strategies, and exposure to domestic violence (lifetime, during pregnancy, and in the past year). Women with traumatic childbirth experiences had a 14.7-fold higher risk of developing P-PTSD. Lifetime trauma, particularly physical or sexual abuse and exposure to disasters, further increased vulnerability. Over the last three months, 50% of those initially diagnosed continued to meet the diagnostic criteria. Multivariate analysis identified traumatic childbirth, state anxiety, and domestic violence during pregnancy as significant predictors of postpartum post-traumatic stress. **Conclusions**: Postpartum post-traumatic stress is a significant and underestimated consequence of high-risk deliveries. Screening for domestic violence and trauma history during pregnancy, assessing perinatal anxiety, and providing trauma-informed psychological support are critical to reducing maternal psychiatric morbidity and promoting maternal-infant well-being.

## 1. Introduction

Childbirth is traditionally regarded as a positive and life-affirming event. However, for a significant proportion of women, the experience can be perceived as threatening, painful, or traumatic, leading to lasting psychological consequences [1].

Post-Traumatic Stress Disorder (PTSD) is a mental health condition characterized by intrusive memories, avoidance behaviors, negative alterations in cognition and mood, and hyperarousal following exposure to a traumatic event [2]. According to the Diagnostic and Statistical Manual of Mental Disorders (DSM-5) [3], PTSD may arise after events involving actual or threatened death, serious injury, or sexual violence—whether directly experienced, witnessed, learned about or as a continuous exposure to the trauma of workplace.

Postpartum PTSD (P-PTSD) refers to PTSD symptoms developing after childbirth, often when labor or delivery is perceived as life-threatening for the mother or faetus/neonate. However, the prevalence of P-PTSD in the general postpartum population is estimated to be between 1.5% and 6% [4], but rates rise dramatically in high-risk obstetric scenarios. Emergency Caesarean Section (EMCS) is associated with increased likelihood of maternal distress due to the invasive nature of the procedure, unexpected medical complications, and the sense of loss of control during delivery [5,6]. Prior studies have shown that approximately one in four women undergoing EMCS develops P-PTSD [7], while the risk following traumatic vaginal birth remains less well documented.

Beyond the delivery method, several psychosocial factors contribute to the onset of P-PTSD. More specifically, domestic violence has consistently been linked to adverse perinatal mental health outcomes, including depression, anxiety, and PTSD [8]. In addition, domestic violence during pregnancy not only jeopardizes maternal safety, but also increases obstetric complications and negatively impacts neonatal health [9]. Furthermore, a history of traumatic life events—such as childhood abuse, assault, or severe accidents—can sensitize individuals to stress responses, heightening vulnerability to PTSD when exposed to new traumatic stimuli such as a complicated birth [10,11], Personality traits, particularly high trait anxiety, and maladaptive coping strategies have also been implicated as risk factors according to the bibliography [12].

From a nursing and midwifery perspective, understanding P-PTSD is essential for developing trauma-informed models of perinatal care. Nurses and midwives are often the first professionals to observe early signs of psychological distress after childbirth. Their role in screening, providing emotional support, and facilitating referrals to mental health services is therefore critical. By identifying psychosocial and obstetric predictors of P-PTSD, this study contributes to evidence-based nursing practice aimed at improving maternal mental health outcomes and promoting safe, respectful maternity care. Despite a growing body of literature on P-PTSD, few studies have prospectively examined the combined effect of obstetric risk, violence, lifetime traumas, and personality characteristics within the same study. Understanding these associations is crucial for identifying high-risk women early and implementing preventive mental health interventions. So, this prospective study aimed to determine the prevalence of P-PTSD among women undergoing EMCS or Operative Vaginal Delivery (OVD), as well as the contribution of violence, lifetime trauma history, personality characteristics (trait anxiety and coping strategies), obstetric variables and socio-demographic factors in the development of P-PTSD. Our research hypotheses were: (a) women with higher levels of state and trait anxiety, exposure to domestic violence, and a history of lifetime trauma would be more likely to develop P-PTSD following a traumatic birth, (b) positive coping strategies would serve as protective factors against P-PTSD, (c) traumatic birth experiences (EMCS or OVD) would independently predict P-PTSD at six weeks and three months postpartum.

## 2. Materials and Methods

This prospective study was designed and reported according to the STROBE (Strengthening the Reporting of Observational Studies in Epidemiology) guidelines to ensure methodological transparency and completeness. It was conducted between March and July 2023 in the maternity clinics of two tertiary hospitals in Athens, Greece: “Elena Venizelou” General Hospital and “Alexandra” General Hospital. The study was approved by the respective institutional ethics committees (Elena Venizelou: Protocol No. 003, approval date 22 March 2023; Alexandra: Protocol No. 562, approval date 23 September 2022) and adhered to the principles of the Declaration of Helsinki. All participants provided written informed consent prior to enrollment.

However, given the sensitive nature of the questions on trauma and violence, several protective measures were implemented: (a) participation was voluntary and anonymous; (b) participants could withdraw at any stage without consequences; (c) questionnaires were completed in a private room to ensure confidentiality; (d) emotional support was available through the hospital’s psychological services, and women who reported distress or domestic violence were offered immediate referral to a mental health professional and finally (e) all data were securely stored in password-protected files accessible only to the research team.

### 2.1. Participants

Eligible participants were postpartum women who had delivered via EMCS or OVD. These delivery modes were selected due to their established association with higher psychological distress and trauma risk. The inclusion criteria were: (a) age ≥ 18 years, and (b) delivery by EMCS or OVD. Therefore, the exclusion criteria were: (a) cognitive impairment or inability to complete questionnaires, (b) current use of psychotropic substances and (c) inability to understand and communicate in Greek.

Participation in the study was entirely voluntary, and women retained the right to withdraw at any stage without providing a reason and without any impact on the care they received. Women were excluded from further participation if they declined to provide informed consent at recruitment or if they later withdrew their consent during follow-up. In cases where consent was withdrawn, no additional data were collected and any identifiable information was immediately removed from the study records. Only data collected prior to withdrawal were retained in anonymised form in accordance with ethical guidelines.

#### Sample Size Calculation

The sample size was calculated for the logistic regression method [13,14]. Assuming an effect size of f = 0.15, α = 0.05, and power = 0.80, a minimum of 90 participants was required.

### 2.2. Procedure

A convenience sampling method was used. Eligible postpartum women who were present in the maternity wards during the data collection period were approached and informed about the study. While recruitment was not strictly consecutive, all eligible women available at the time of data collection were consistently invited to participate in order to reduce selection bias during the data collection period (March–July 2023). Women were informed about the study on the second postpartum day, after they had been transferred to the postnatal ward and were clinically stable. At that time, they received verbal and written information regarding the aims, procedures, confidentiality, and voluntary nature of participation, and were given the opportunity to ask questions before providing written informed consent. The researchers informed potential participants about the study objectives, assured confidentiality, and invited them to participate voluntarily. Those who met the inclusion criteria and provided written informed consent were enrolled. Recruitment continued until the desired sample size was reached. Although the a priori sample size calculation indicated that a minimum of 90 participants was required, recruitment continued until 113 women were enrolled. This was performed to compensate for potential loss to follow-up at the 6-week and 3-month assessments, which is common in longitudinal postpartum research. Additionally, a slightly larger sample enhances the precision of estimates and strengthens the statistical power of multivariable analyses, especially for outcomes with relatively low prevalence such as P-PTSD.

### 2.3. Data Collection

The data were collected by the research team of midwives trained in trauma-informed research methods. Data collection took place in the postnatal wards of the two hospitals. Eligible participants were identified from hospital delivery records and approached on the second postpartum day. After receiving detailed information about the study, those who consented completed the questionnaires under the guidance of the researchers. Follow-up assessments at six weeks and three months postpartum were conducted via telephone interviews with participants. The follow-up assessments at 6 weeks and 3 months were conducted via telephone interviews. Data were collected at three time points.

Stage 1—Second postpartum day:

- Socio-demographic and medical data form: Included age, education, marital and employment status, income, obstetric history, and relevant medical/psychiatric history.
- Abuse Assessment Screen (AAS) [15] and a Greek-developed questionnaire to assess lifetime and pregnancy-specific violence exposure [16]. The AAS is a 5-item validated screening tool designed to detect physical, sexual, and psychological violence during a woman’s lifetime, within the past year, and during pregnancy. The Greek validation demonstrated satisfactory internal consistency (Cronbach’s α = 0.80), strong criterion validity against clinical interviews, and high sensitivity for detecting intimate partner violence. The AAS begins with a general question about lifetime abuse and proceeds with branching questions based on positive responses, enabling identification of type, timing, and perpetrator of violence. In this study, the AAS functioned as the primary structured measure for domestic violence screening, while the supplementary Greek questionnaire captured culturally specific experiences of abuse not covered by the original AAS.
- Trauma History Questionnaire (THQ) [17]. A 24-item self-report tool from the U.S. National Center for PTSD assessing exposure to various traumatic events. The THQ is a structured checklist composed of 24 yes/no items covering three domains of traumatic exposure: (a) crime-related events, (b) general disasters and accidents, and (c) physical or sexual trauma. For each endorsed event, respondents indicate the frequency and the age at which the trauma occurred, allowing the instrument to capture both the timing and the developmental context of exposure. The THQ functions as a lifetime trauma inventory rather than a severity scale, and higher number of endorsed events reflects greater cumulative trauma exposure. The tool has been widely used in clinical and community samples internationally and has demonstrated high internal consistency (Cronbach’s α = 0.86), good test–retest reliability, and strong convergent validity with other trauma measures. Although no formal Greek validation study exists, the THQ has been used extensively in Greek research on trauma and perinatal mental health, and its content is compatible with the cultural and clinical context of the present study. The Greek version used in this research followed standard translation procedures (forward–backward translation) and was pilot-tested for clarity and comprehension prior to data collection.
- State-Trait Anxiety Inventory (STAI-40) [18]. Measures current state anxiety and trait anxiety. The questionnaire was designed by Spielberger C.D. and adapted into Greek by Liakos A. and Giannitsis S [19].
- Coping Strategies Questionnaire (CSQ-38). Evaluates coping mechanisms, including positive reappraisal, problem solving, social support seeking, metaphysical coping, avoidance, and confrontation. It was adapted into Greek by Karadimas in 1998, from whom permission to use it was obtained [20]. The CSQ-38 is a structured self-report instrument consisting of 38 items rated on a 5-point Likert scale, assessing the frequency with which individuals use different coping strategies. Items are grouped into six subscales: positive reappraisal, problem solving, social support seeking, metaphysical coping, avoidance, and confrontation. Subscale scores are obtained by summing the relevant items, with higher scores indicating greater use of the corresponding coping style. The questionnaire captures both adaptive and maladaptive coping mechanisms, providing a multidimensional profile of coping responses. The Greek adaptation by Karadimas (1998) [21] demonstrated satisfactory psychometric properties, including acceptable internal consistency (Cronbach’s α = 0.78 across subscales), stable factorial structure, and good construct validity in Greek adult populations. The CSQ-38 has been widely used in Greek psychological and health-related research and is considered appropriate for assessing coping in perinatal populations.
- Criterion A of P-PTSD: Questions tailored to EMCS and OVD scenarios to determine exposure to potentially traumatic childbirth events. More specifically, to determine whether participants met Criterion A for P-PTSD, a structured questionnaire was developed based on the DSM-5 criteria for exposure to a traumatic event and tailored specifically to the two clinical scenarios investigated: EMCS and OVD. The measure assessed whether the woman experienced childbirth as (a) actual or threatened death or serious injury to herself or her child, (b) a feeling of extreme fear or helplessness during labor or surgery, and (c) complications for herself or her child after labor or surgery. For EMCS, the data explored perceptions of sudden clinical deterioration, emergency transfer to the operating room, and fear of harm to the mother or fetus/neonate. For OVD, the items assessed perceptions of invasive intervention, unexpected complications, and concerns about fetal distress. Responses were recorded on a dichotomous basis (yes/no), and endorsement of any exposure meeting DSM-5 criteria constituted satisfaction of Criterion A. This approach follows established methods in perinatal PTSD research and ensures consistent recognition of traumatic exposures related to childbirth across all modes of delivery.

Stage 2—Six weeks postpartum:

- PTSD Checklist for DSM-5 (PCL-5) [22]. Assesses the presence and severity of PTSD symptoms across four DSM-5 clusters (B–E). A Greek adaptation with strong psychometric properties in postpartum populations was used [23]. The Greek adaptation of the PTSD Checklist for DSM-5 (PCL-5) has demonstrated excellent psychometric performance in postpartum populations. Internal consistency is very high, with Cronbach’s α values typically ranging from 0.90 to 0.94 for the total scale and from 0.78 to 0.91 across the four DSM-5 symptom clusters (intrusion, avoidance, negative alterations in cognition and mood, and arousal). Studies in Greek perinatal samples have confirmed strong convergent validity with established measures of anxiety, depression, and traumatic stress, as well as robust discriminant validity. The scale also shows excellent diagnostic accuracy, with sensitivity and specificity values above 0.85 when recommended cut-off scores for probable PTSD. Its factor structure has been shown to be stable and consistent with DSM-5 symptom clusters. In this study, scoring and interpretation followed the Greek validation guidelines, with higher scores indicating greater symptom severity and a cut-off of ≥31 or meeting the criteria used to identify probable postpartum PTSD.

Stage 3—Three months postpartum:

- Reassessment of P-PTSD symptoms via PCL-5 for women meeting PTSD criteria at Stage 2.

### 2.4. Statistical Analysis

Data were analyzed using IBM SPSS Statistics (version 27). Descriptive statistics summarized sociodemographic and obstetric characteristics. Means and standard deviations were calculated for normally distributed continuous variables, while medians and interquartile ranges were used for non-normally distributed variables. Categorical sociodemographic and obstetric characteristics were summarized using frequencies and percentages. Chi-square (χ^2^) tests for independency are used in order to evaluate dependency between categorical characteristics and P-PTSD. Independent sample t-test is used in order to compare continuous characteristics between woman with P-PTSD and woman without P-PTSD. Finally, multivariate analysis was performed to investigate which variables are important in predicting the diagnosis of P-PTSD in postpartum women. For this purpose, the multiple logistic regression method was applied using Forward: LR criterion. Odds ratios and the corresponding 95% Confidence Interval (95% CI) calculated. Statistical significance was set at *p* < 0.05. Although stepwise methods, including Forward: LR, have recognized limitations such as potential model instability and risk of overfitting, this approach was selected because the study was exploratory and aimed to identify the most parsimonious set of predictors associated with P-PTSD. To reduce these limitations, all variables entered in the stepwise model had theoretical justification based on prior literature, and model diagnostics were performed to ensure acceptable fit and stability.

## 3. Results

### 3.1. Participant Characteristics

Of the 113 women included in the study, 73.5% (n = 83) delivered via EMCS and 26.5% (n = 30) via OVD. The majority were married (89.4%), of Greek nationality (83.2%), and university graduates (53.1% held a bachelor’s degree or higher). Nearly one-third (31%) were unemployed at the time of delivery, and 30.1% reported no personal income. Almost half (48.7%) had no prior childbirth experience. A history of psychiatric illness was reported by 11.5%, while 15.9% had a significant medical history.

At six weeks postpartum, 30.1% met Criterion B, 22.1% met Criterion C, 21.2% met Criterion D, and 20.5% met Criterion E for PTSD according to DSM-5. Overall, 14.2% of the sample met the full diagnostic criteria for PTSD at this stage.

### 3.2. Association Between Anxiety and P-PTSD

The results of the analysis showed that there is a statistically significant difference in both state anxiety (t = −5.510, *p* = 0.000) and anxiety as a personality trait (t = −3.994, *p* = 0.000). The findings show that women who developed P-PTSD (MO = 53.8, SD = 13.5) had significantly higher levels of state anxiety compared to women who did not develop P-PTSD (MO = 36.7, SD = 11.1). Similarly, women who developed P-PTSD (MO = 49.6, SD = 11.4) had significantly higher levels of anxiety as a personality trait compared to women who did not develop P-PTSD (MO = 38.5, SD = 10.1) (Table 1).

### 3.3. Association Between Coping Strategies and P-PTSD

The next analysis aimed to assess whether there was a significant difference in coping strategies between women who developed P-PTSD and women who did not. The t-test for two independent groups was used for this test, and the findings are presented in Table 2. The results of the analysis showed that there is a statistically significant difference in coping strategies: positive reappraisal (t = 2.873, *p* = 0.005), problem solving (t = 2.214, *p* = 0.029), and positive coping strategies (t = 2.971, *p* = 0.004). The findings show that women who did not develop P-PTSD use coping strategies associated with positive reappraisal (2.1 ± 0.4 vs. 1.8 ± 0.4), problem solving (1.9 ± 0.5 vs. 1.6 ± 0.6), and positive coping strategies (2.0 ± 0.4 vs. 1.7 ± 0.4), compared to women who developed P-PTSD (Table 2).

### 3.4. Association Betweenviolence and P-PTSD

The study also investigated whether there is a link between experiences of domestic violence and P-PTSD. The chi-square independence test revealed a statistically significant dependence of the occurrence of P-PTSD on experiences of domestic violence (χ^2^ = 23.403, *p* = 0.000), the type of violence (χ^2^ = 23.680, *p* = 0.000), domestic violence in the last year (χ^2^ = 15.177, *p* = 0.000), domestic violence during pregnancy (χ^2^ = 17.132, *p*= 0.000) and women’s fear of their partner (χ^2^ = 21.080, *p* = 0.000) (Table 3).

### 3.5. Traumatic Experiences

#### 3.5.1. Lifetime Trauma and P-PTSD

Table 4 shows that there is a significant correlation between P-PTSD and women who had experienced trauma from natural disasters (χ^2^ = 7.451, *p* = 0.006), the age at which they experienced natural disasters and trauma (χ^2^ = 8.851, *p* = 0.012), and whether women had experienced traumatic physical and sexual experiences (χ^2^ = 12.372, *p* = 0.000), with the age at which they had experienced traumatic physical and sexual experiences (χ^2^ = 16.945, *p* = 0.000). The results show that among women with P-PTSD, the majority had experienced trauma from general disasters (68.8% vs. 33.0%) and had experienced traumatic physical and sexual experiences (38.5% vs. 7.2%). Similarly, the analysis showed that among women with P-PTSD, a higher percentage had experienced general disasters at the age of over 18 (56.3%) and a higher percentage had experienced traumatic physical and sexual experiences under the age of 18 (31.3%) (Table 4).

#### 3.5.2. Traumatic Birth Experience

**Figure 1:**
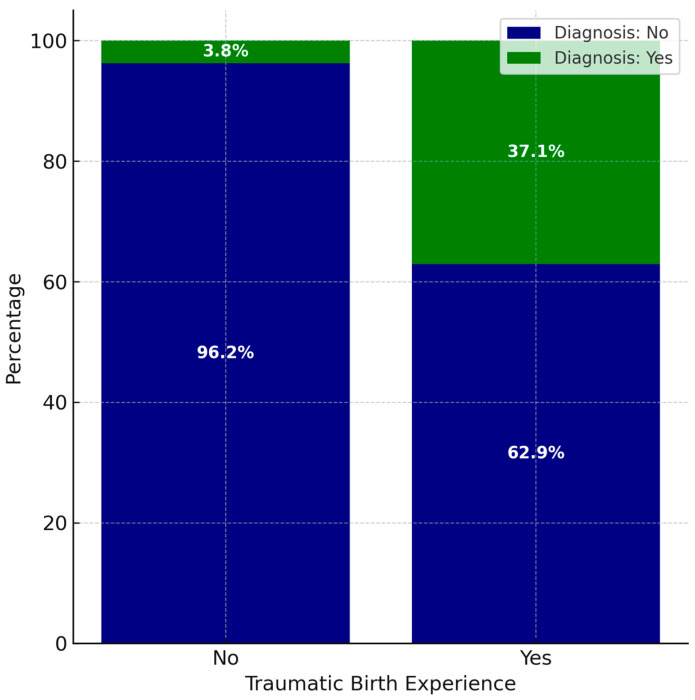
Percentages of women with or without diagnosis and birth experiences.

In addition, the findings show that of the women who experienced a traumatic event during childbirth, 37.1% developed P-PTSD. In contrast, among women who did not experience any traumatic event during childbirth, 3.8% developed P-PTSD (Figure 1). Results indicate that the presence of a traumatic experience during childbirth is associated with a 14.7 greater likelihood of developing P-PTSD (χ^2^ (1) = 20.561, *p* < 0.001, OR = 14.7, 95% CI = [3.9, 56.5]) (Figure 1).

### 3.6. P-PTSD 3 Months After the First Assessment

Of the 16 women who initially presented with P-PTSD, 14 were reassessed three months after the initial assessment. More specifically, from the 14 women, 7 (50%) still had P-PTSD 3 months after the initial assessment.

### 3.7. Multivariate Logistic Regression Analysis

Below are the findings of the statistical analysis conducted to investigate which variables are important in predicting P-PTSD. For this purpose, the multiple logistic regression method was applied using the forward variable introduction methodology with the Forward: LR criterion. The multivariate analysis shows that the characteristics that are important in predicting the onset of P-PTSD are traumatic experiences during childbirth (b = −2.010, *p* = 0.009, Exp (B) = 0.122, 95% CI = [0.026, 0.586]), situational stress (b = 0.078, *p* = 0.006, Exp (B) = 1.082, 95% CI = [1.023, 1.144]) and domestic violence during pregnancy (b = −2.562, *p* = 0.011, Exp (B) = 0.077, 95% CI = [0.011, 0.559]). These characteristics can accurately predict the presence or absence of P-PTSD in 90.3% of cases. These results show that an increase in state anxiety by one unit is associated with an 8.2% increase in the likelihood of developing P-PTSD. Similarly, the results show that women who experienced traumatic events during childbirth are 8.2 times more likely to develop P-PTSD than women who did not experience traumatic events. Finally, the analysis shows that women who experienced domestic violence during pregnancy are 12.9 times more likely to develop P-PTSD than women who did not experience domestic violence during that period (Table 5).

## 4. Discussion

This study aimed to investigate the prevalence of P-PTSD in high-risk groups of women and to explore personality traits, domestic violence, and history of psychological trauma as mediators in the development of P-PTSD. The study’s findings showed that traumatic experience before, during, and after childbirth (criteria A), the mother’s anxiety on the second day of postpartum, domestic violence during pregnancy, and anxiety as a condition significantly predict the onset of PTSD. There are many studies linking stress to P-PTSD. It is a fact that a diagnostic feature of PTSD and anxiety disorders is the dysregulation of the brain circuits that mediate fear responses [24]. Anxiety is the first reaction to trauma, and sensitivity to anxiety is a risk factor for developing PTSD, due to the conceptual identification of the individual’s perception of trauma and the high rate of comorbidity among individuals suffering from PTSD [25]. Furthermore, criterion A, is required according to DSM-5 in order to diagnose PTSD [26]. In this particular study, criterion A concerned exposure to imminent death or threat to the life of the mother or neonate and was determined by specific questions that explored this feeling of extreme fear of losing one’s life. On the other hand, domestic violence is a factor in the development of PTSD, since fear of loss of life and abuses are criteria A for the development of PTSD [27]. It seems that constant exposure to violence within the family environment can affect a woman’s ability to process and cope with these experiences, resulting in chronic stress that triggers the development of P-PTSD. Additionally, domestic violence during pregnancy is associated with increased complications for the mother and fetus/neonate [8].

The findings of this study also are consistent with previous research showing that anxiety, prior trauma, and domestic violence are among the strongest predictors of postpartum PTSD. Several studies [28,29,30] similarly report elevated rates of P-PTSD following emergency caesarean section or operative delivery, emphasizing the role of perceived threat and loss of control. Moreover, longitudinal research suggests that the persistence of symptoms beyond three months postpartum, as observed here, highlights the need for ongoing psychosocial monitoring and targeted interventions [31].

The implications for nursing and midwifery practice are substantial. Integrating trauma-informed approaches into perinatal care can help reduce women’s fear and enhance their sense of safety during childbirth. Nurses and midwives can play a pivotal role in early screening for domestic violence, anxiety, and previous trauma during antenatal visits, using validated tools such as the AAS and PCL-5. Training healthcare professionals to recognize signs of postpartum stress and provide empathic, evidence-based support may prevent chronic psychiatric morbidity and improve both maternal and neonatal outcomes. The implementation of psychosocial risk assessment protocols within maternity units could thus serve as a preventive measure, aligning with WHO recommendations for holistic maternal care.

This study has several notable strengths. First, it employed a prospective design, with data collection at multiple time points (early postpartum, 6 weeks, and three months). This approach allowed for temporal assessment of P-PTSD symptom trajectories, minimizing recall bias. Second, the study combined obstetric, psychosocial, and psychological variables (including domestic violence, lifetime trauma, anxiety, and coping strategies) providing a comprehensive perspective on P-PTSD risk factors. Third, validated and culturally adapted instruments were used (e.g., STAI, PCL-5, CSQ-38), ensuring reliability of the psychometric assessments. Additionally, the focus on a Greek maternity population adds valuable data from a cultural context that remains underrepresented in the international literature, because in Greece the rates of CS are high and increasing, with minimal rates of OVD. Additionally, this study followed the STROBE recommendations, ensuring methodological rigor and transparent reporting across all stages of design, data collection, and analysis. However, several limitations should be acknowledged. The sample size (n = 113) was relatively modest and limited to two tertiary hospitals in Athens, which may restrict generalizability to other settings, particularly rural populations. Second, although prospective, the follow-up period extended only to 3 months postpartum, preventing conclusions about long-term persistence of P-PTSD symptoms. Third, data on violence and trauma history relied on self-report questionnaires, which may be affected by underreporting due to stigma, fear, or recall bias. Fourth, multiple bivariate tests were conducted across several psychosocial and obstetric variables, which increases the risk of Type I error (false positive findings). However, the main conclusions of the bivariate analyses should be interpreted with caution. Finally, as an observational, uncontrolled study, the present research cannot establish causal relationships, and the findings should be interpreted as associations rather than evidence of causation.

## 5. Conclusions

P-PTSD is an important but underestimated consequence of high-risk births. Our study highlights the interaction of stress, exposure to violence, and traumatic experiences during childbirth and coping mechanisms in shaping women’s vulnerability to P-PTSD. The findings underscore the need for: (a) systematic screening for domestic violence and prior trauma during pregnancy, (b) routine assessment of anxiety among women undergoing emergency situations, and (c) targeted interventions, such as trauma-informed care, psychological support, and coping skills training to mitigate the risk of long-term psychiatric morbidity. Addressing P-PTSD is critical not only for maternal well-being but also for infant development and family health. Integrating psychosocial assessments into perinatal care pathways may significantly reduce the burden of postpartum trauma and enhance maternal recovery.

## Figures and Tables

**Table 1:** Anxiety Scores by P-PTSD Status.

Anxiety Measure	No P-PTSD (Mean ± SD)	P-PTSD (Mean ± SD)	t	*p*
State Anxiety (STAI-State)	36.7 ± 11.1	53.8 ± 13.5	−5.510	0.000
Trait Anxiety (STAI-Trait)	38.5 ± 10.1	49.6 ± 11.4	−3.994	0.000

**Table 2:** Coping Strategies by P-PTSD Status.

Coping Strategy	No P-PTSD (Mean ± SD)	P-PTSD (Mean ± SD)	t	*p*
Positive re-evaluation	2.1 ± 0.4	1.8 ± 0.4	2.873	0.005
Problem solving	1.9 ± 0.5	1.6 ± 0.6	2.214	0.029
Positive coping strategies	2.0 ± 0.4	1.7 ± 0.4	2.971	0.004
Social support	2.0 ± 0.6	1.9 ± 0.5	0.498	0.620
Wishful thinking	1.7 ± 0.7	1.5 ± 0.6	0.617	0.538
Wishful thinking (metaphysical sources)	1.6 ± 0.9	1.4 ± 0.9	0.770	0.443
Avoidance-escape/resignation	1.5 ± 0.7	1.4 ± 0.5	0.076	0.940
Refusal	1.4 ± 0.7	1.2 ± 0.7	1.488	0.140
Assertive problem solving	1.4 ± 0.6	1.2 ± 0.7	1.172	0.244

**Table 3:** Domestic Violence and P-PTSD.

		Νο P-PTSD (n (%))	P-PTSD (n (%))	χ^2^	*p*
Domestic violence in your life	No	82 (84.5%)	5 (31.3%)	23.403	0.000
	Yes, from my parents when I was child	4 (4.1%)	4 (25.0%)		
Type of violence	Yes, from my partner	7 (7.2%)	7 (43.8%)		
	From my ex	4 (4.1%)	0 (0.0%)		
	None	83 (85.6%)	5 (31.3%)	23.680	0.000
	Psychological	8 (8.2%)	2 (12.5%)		
	Physical	1 (1.0%)	1 (6.3%)		
	Both	5 (5.2%)	8 (50%)		
Domestic violence last year	No	92 (94.8%)	9 (56.3%)	15.177	0.000
	Yes	5 (5.2%)	7 (43.8%)		
Domestic violence during pregnancy	No	95 (97.9%)	10 (62.5%)	17.132	0.000
	Yes	2 (2.1%)	6 (37.5%)		

**Table 4:** P-PTSD and life events.

		Νο P-PTSD (n (%))	P-PTSD (n (%))	χ^2^	*p*
Crime-related incidents	No	55 (56.7%)	10 (62.5%)	0.189	0.664
	Yes	42 (43.3%)	6 (37.5%)		
Crime-related incidents (age)	No	55 (56.7%)	10 (62.5%)	2.980	0.225
	<18	10 (10.3%)	3 (18.8)		
	>18	32 (33.0%)	3 (18.8%)		
General disasters and traumas	No	65 (67.0%)	5 (31.3%)	7.451	0.006
	Yes	32 (33.0%)	11 (68.8%)		
General disasters and traumas (age)	No	65 (67.0%)	5 (31.3%)	8.851	0.012
	<18	4 (4.1%)	2 (12.5)		
	>18	28 (28.9%)	9 (56.3%)		
Physical and sexual experiences	No	90 (92.8%	10 (62.5%)	12.372	0.000
	Yes	7 (7.2%)	6 (37.5)		
Physical and sexual experiences (age)	No	90 (92.8%)	10 (62.5%)	16.945	0.000
	<18	3 (3.1%)	5 (31.3%)		
	>18	4 (4.1%)	1 (6.3%)		

**Table 5:** Multivariate logistic regression with P-PTSD onset as the dependent variable.

95%C.I.									
		B	S.E.	Wald	df	Sig.	Exp(B)	L	U
Step 1	Domestic violence during pregnancy	−3.350	0.882	14.439	1	0.000	0.035	0.006	0.197
	Constant	1.099	0.816	1.810	1	0.178	3.000		
Step 2	State Anxiety	0.099	0.029	11.861	1	0.001	1.104	1.043	1.167
	Domestic violence during pregnancy	−2.748	1.048	6.876	1	0.009	0.064	0.008	0.500
	Constant	−3.837	1.733	4.899	1	0.027	0.022		
Step 3	Traumatic birth experience	−2.101	0.799	6.914	1	0.009	0.122	0.026	0.586
	State anxiety	0.078	0.028	7.597	1	0.006	1.082	1.023	1.144
	Domestic violence during pregnancy	−2.562	1.010	6.432	1	0.011	0.077	0.011	0.559
	Constant	−2.120	1.713	1.531	1	0.216	0.120		

## Data Availability

The data is available upon request.

## Public Involvement Statement

No public involvement in any aspect of this research.

## Guidelines and Standards Statement

This manuscript was drafted against the STROBE guidelines.

## Abbreviations

The following abbreviations are used in this manuscript:

PTSD	Posttraumatic Stress Disorder
P-PTSD	Postpartum Posttraumatic Stress Disorder
EMCS	Emergency Caesarean Section
OVD	Operative Vaginal Delivery
